# Loss of reproductive output caused by an invasive species

**DOI:** 10.1098/rsos.150481

**Published:** 2016-04-06

**Authors:** Maude E. M. Tremblay, Todd J. Morris, Josef D. Ackerman

**Affiliations:** 1 Department of Integrative Biology, University of Guelph, Guelph, Ontario, CanadaN1G 2W1; 2 Great Lakes Laboratory for Fisheries and Aquatic Sciences, Fisheries and Oceans Canada, 867 Lakeshore Road, Burlington, Ontario, CanadaL7S 1A1

**Keywords:** unionid mussels, species at risk, *Neogobius melanostomus*, Great Lakes

## Abstract

We investigated whether *Neogobius melanostomus*, an invader of biodiversity ‘hot-spots’ in the Laurentian Great Lakes region, facilitates or inhibits unionid mussel recruitment by serving as a host or sink for their parasitic larvae (glochidia). Infestation and metamorphosis rates of four mussel species with at-risk (conservation) status (*Epioblasma torulosa rangiana*, *Epioblasma triquetra*, *Lampsilis fasciola* and *Villosa iris*) and one common species (*Actinonaias ligamentina*) on *N. melanostomus* were compared with rates on known primary and marginal hosts in the laboratory. All species successfully infested *N. melanostomus,* but only *E. triquetra, V. iris* and *A. ligamentina* successfully metamorphosed into juveniles, albeit at very low rates well below those seen on even the marginal hosts. *Neogobius melanostomus* collected from areas of unionid occurrence in the Grand and Sydenham rivers (Ontario, Canada) exhibited glochidial infection rates of 39.4% and 5.1%, respectively, with up to 30 glochidia representing as many as six unionid species per fish. A mathematical model suggests that *N. melanostomus* serve more as a sink for glochidia than as a host for unionids, thereby limiting recruitment success. This represents a novel method by which an invasive species affects a native species.

## 1. Introduction

The Laurentian Great Lakes have experienced a number of threats and impacts including those from invasive dreissenid mussels in the late 1980s [1,2], which extirpated native unionid mussels by interfering with their locomotion and burrowing and preventing them from closing their valves [3]. Although unionids remain in some tributary and embayment refugia of the Great Lakes [4–6], 15 of 41 unionid species in Ontario have been assessed as at risk for extinction or extirpation by the Committee on the Status of Endangered Wildlife in Canada [6]. Several large rivers in southwestern Ontario are considered species conservation ‘hot-spots' [5] because of the high diversity of unionids present. The recent expansion of the round goby (*Neogobius melanostomus*) into the lower reaches of these rivers is cause for concern [6,7] because of the potential for predation on juvenile unionids and competition with and/or predation on native host fishes required by unionids for their larval development [6,8,9]. However, such assessments have not considered whether unionid mussels can use *N. melanostomus* as hosts to complete their life cycle and mitigate these hypothesized impacts.

Unionids have an obligate parasitic larval stage (glochidium), which requires a vertebrate host (usually a fish) to facilitate metamorphosis into free-living juvenile mussels [10,11]. Among unionids, there are host generalists (able to use many host fishes) and host specialists (only use a few host fish species) and many species have evolved elaborate species-specific strategies of host attraction [9]. For a fish to be considered a host, both glochidial infection and metamorphosis must occur; primary hosts have high infestation rates (intensity of infection or the proportion of glochidia that attach) and high metamorphosis rates (proportion of attached glochidia that metamorphose into juveniles), whereas marginal hosts provide lower rates, particularly metamorphosis rates, for a given mussel species [11]. *Neogobius melanostomus* may be a marginal host for several unionid species in the laboratory [12] (J.D.A. 2010, personal communication) and glochidial parasitism was reported for a single individual in the field [13]. Given the capacity for high post-invasion abundance of *N. melanostomus* [14], *N. melanostomus* may either facilitate the recruitment of unionid mussels as a host fish or limit successful unionid recruitment by removing large proportions of glochidia through initial attachment followed by low or unsuccessful metamorphosis (i.e. serving as a sink for glochidia) as observed for non-host species [15]. This may contribute to the biotic homogenization of the region, which can decrease the availability of native host fishes to mussels [16]. The role of *N. melanostomus* as a host for unionids or as a sink for their glochidia remains to be determined.

The purpose of this study is to determine whether *N. melanostomus* serve as hosts for unionid mussels or as sinks for their glochidia. As hosts, they should facilitate glochidial infection and metamorphosis of large numbers of juvenile mussels, and thus propagate unionids. As sinks, glochidia should attach but not metamorphose (or do so at low rates), thereby reducing unionid mussel recruitment. We investigated the host quality of *N. melanostomus* in the laboratory using controlled infection experiments, and in the field where natural glochidia infections of *N. melanostomus* were evaluated to determine the abundance and species of glochidia.

## 2. Material and methods

### 2.1. Assessment of infection and metamorphosis on *Neogobius melanostomus* in the laboratory

Gravid female *Epioblasma torulosa rangiana* (Lea 1838) (an endangered species), and *Actinonaias ligamentina* (Lamarck 1819) (a common species) were collected from the Sydenham River at Florence, Ontario (42.655677, −82.008247) and *Epioblasma triquetra* (Rafinesque 1820) (an endangered species) were collected at Dawn Mills, Ontario (42.600609, −82.12635). *Lampsilis fasciola* Rafinesque 1820 (a species of special concern) and *Villosa iris* (Lea 1829) (an endangered species) were collected from the Thames River at Thamesford, Ontario (43.05601, −80.99274). Three females of each species and similar size were transported in aerated coolers to the Hagen Aqualab (University of Guelph) where they were acclimated to 16–18°C and moved to a circular flow-through tank containing well water at approximately 11°C. Mussels were fed an algal diet (2 × 10^8^ cells l^−1^ of Nanno 3600 and shellfish diet, Reed Mariculture, Campbell, CA, USA) three times per week.

Young-of-the-year fish, whenever possible, were collected from water bodies in Guelph, Burlington, Dundas, and Niagara Falls, Ontario that did not contain the mussel species of interest (i.e. to reduce immunological responses due to previous glochidial encystment [17]). Species included *N. melanostomus* (Pallas, 1814), *Cottus bairdii* Girard, 1850, a known marginal host for all mussel species and ecologically similar to *N. melanostomus* [9,18], and primary fish hosts of the respective mussel species (*Micropterus salmoides* (Lacepède, 1802) for *A. ligamentina*; *Etheostoma exile* (Girard, 1859) for *E. t. rangiana*; *Percina caprodes* (Rafinesque, 1818) for *E. triquetra*; *Micropterus dolomieu* Lacepède, 1802 for *L. fasciola* and *Ambloplites rupestris* (Rafinesque, 1817) for *V. iris*). Fish were transported in aerated buckets and acclimated to 16–18°C prior to use in experiments (2010) or were treated with Melafix (Mars Fishcare, Chalfont, USA; 0.125 ml l^−1^ tea tree oil) for 3 days and quarantined for at least one week prior to experiments (2011). Fish were maintained on bloodworms, crayfish, brine shrimp and/or smelt (Metro Ontario, Inc.) as appropriate several times weekly.

Laboratory infections, which followed the techniques in [11], provided an opportunity to evaluate the capacity for successful host-glochidia infection among fish species in a controlled manner regardless of the mechanism used by a particular mussel species (e.g. broadcasting, host attraction, direct contact) [9]; it was not designed to estimate infection rates by drifting glochidia rather it is closer to that of host attraction and direct contact strategies given the concentration of glochidia used. Briefly, glochidia from half the gill of each female mussel were assessed for viability [19,20] before being divided into three portions and used to infest four conspecific fish of similar total length (TL) at a concentration of *ca* 5000 glochidia l^−1^ in an aerated 1.0 l tank for 1 h in the dark. Each group of four fish from a specific mussel–fish infestation (i.e. replicate) was placed in one of three Aquatic Habitat (AHAB) units (Pentair Aquatic Habitats, Apopka, USA) operated at 18–20°C using 200 µm-filtered well water. All juvenile mussels and excysted glochidia were removed and counted after being flushed into 100 µm mesh caps located in each AHAB tank (twice weekly) or after $\frac{1}{3}-\frac{1}{2}$ of the water volume of each tank was siphoned through a 100 µm sieve (weekly). Once the recovery caps were devoid of juveniles for seven consecutive days, fish were inspected for glochidial infection under tricaine methanesulfonate (MS-222; approx. 23 mg l^−1^), and if none were found, they were euthanized (MS-222; approx. 100 mg l^−1^) and dissected to confirm the absence of encysted glochidia.

### 2.2. Assessment of natural infection of *Neogobius melanostomus* in the field

*Neogobius melanostomus* were collected, geo-referenced, fixed in formalin and preserved in ethanol from the lower Grand River (between Middleport and Haldimand, Ontario; 43.087610, −80.040690 to 42.958570, −79.869990; June–July 2010) and the East Sydenham River (42.655677, −82.008247 to 42.600609, −82.12635; 16–27 August 2010). The former is slow flowing, deep, and has a substrate composed of clay and mud [20], and the latter is low gradient with a high diversity of habitats (including gravel and sand), exhibits distinct riffles and pools and high turbidity likely resulting from agricultural activities (85% of land use) [21]. A total of 127 *N. melanostomus* from the Grand River (*N* = 127) and 79 from the Sydenham River were measured for TL, the body and fins were examined for glochidial encystment under a 32× dissecting microscope (Nikon SMZ-2 T, Nikon Japan), and glochidial encystment on excised gills was determined using cross polarization. All glochidia were photographed (3.34MP CoolPix 995 Nikon, Japan) and the length, height and hinge length of each glochidium were measured via image analysis (ImageJ 1.38×, US National Institutes of Health).

### 2.3. Statistical analyses

#### 2.3.1. Assessment of infection and metamorphosis on *Neogobius melanostomus* in the laboratory

The infestation rate (*R*_I_) was calculated as the proportion of glochidia that attached to the fish (*G*_A_) from the number used to infest the fish (*G*_T_) (*R*_I_ = *G*_A_/*G*_T_) [11], whereas the metamorphosis rate (*R*_M_) was calculated as the proportion of glochidia that metamorphosed into juvenile mussels (*G*_M_) from *G*_A_ (*R*_M_ = *G*_M_/*G*_A_) [11]. Glochidia encysted on fishes that died prior to the end of the experiment were included in *G*_A_ for determining *R*_I_, but were excluded from *G*_A_ for the determination of *R*_M_.

One-way analysis of variance (ANOVA) was used to compare *R*_I_, *R*_M_ and the number of juveniles produced per fish, respectively, with ‘fish species' as the fixed effect. A two-way main effects ANOVA, with ‘fish species' and ‘female mussel’ as the fixed effects, was used if the viability of glochidia differed among female unionids [18]. Assumption of normality (Shapiro–Wilks test) and homogeneity of variance (Levene's test) were examined and data were ‘arcsine square root’ or ‘log + 1’ transformed as necessary [22]. A non-parametric Kruskall–Wallis test was used if the data were not normal but satisfied homoscedasticity [22]. Significant pairwise treatments were examined using post-hoc Tukey tests [22]. Statistica v. 6.0 (Statsoft, Tulsa, OK, USA) was used for all analyses.

An independent samples *t*-test was used to compare the proportion of fish infected with glochidia (i.e. prevalence of infection) between rivers. A logistic regression was conducted using TL as the predictor and glochidia infection (i.e. 0 = not infected, 1 = glochidia infected) as the dependent variable. All analyses were undertaken using SPSS v. 19 (IBM, Armonk, NY, USA).

#### 2.3.2. Identification of glochidia from the field-collected fish

Glochidia were assigned to species via discriminant function analysis (DFA) [23] using river specific models based on glochidia length, width and hinge length data collected for all 36 species of unionids found in these rivers (*n* = 83 ± 16 (mean ± s.e.) glochidia/species) [24]. Classification success was 78 ± 4% and 75 ± 4% for the Grand and Sydenham rivers, respectively, including five species within two genera that were particularly difficult to resolve [24].

### 2.4. Modelled contribution of *Neogobius melanostomus* to unionid recruitment

A model was used to assess the role of *N. melanostomus* as a host fish for unionids versus a sink for their glochidia using the abundance (*U*) and fecundity (*f;* the number of glochidia per female [25,26] or estimated (i.e. *V. iris*)) of unionids, the encounter rate between glochidia and hosts (*R*_e_; from [27]), and the *R*_I_ and *R*_M_ measured in this study. The infection (*I*_N_) on *N. melanostomus* is given by

2.1 $$I_{N}=U\times f\times R_{e}\times R_{I},$$

whereas the number of juvenile unionid mussels produced (*J*) is given by

2.2 $$\begin{array}{rl} J & =I_{N}\times R_{M}\times N\\ & =(U\times f\times R_{e}\times R_{I})\times R_{M}\times N, \end{array}$$

where *N* is the relative abundance of *N. melanostomus* to known hosts. By extension, the number of glochidia lost from potential recruitment (*D*_g_) is given by

2.3 $$\begin{array}{rl} D_{g} & =I_{N}\times (1-R_{M})\times N\\ & =(U\times f\times R_{e}\times R_{I})\times (1-R_{M})\times N. \end{array}$$

The difference between equations (2.2) and (2.3) is *R*_M_ whereby a higher *R*_M_ leads to more juveniles produced and fewer glochidia lost. The ratio of glochidia loss to juvenile mussel production (*D*_g_ : *J*) for *N. melanostomus* was compared to model results for the primary as well as the marginal host (*C. bairdii*). Although the model is based on parameters derived from controlled laboratory experiments that do not incorporate specific host–fish interactions [9] nor the behaviour of *N. melanostomus*, it does provide an the opportunity to evaluate *D*_g_ : *J*.

## 3. Results

### 3.1. Infection and metamorphosis on *Neogobius melanostomus* in the laboratory

**Figure 1.**
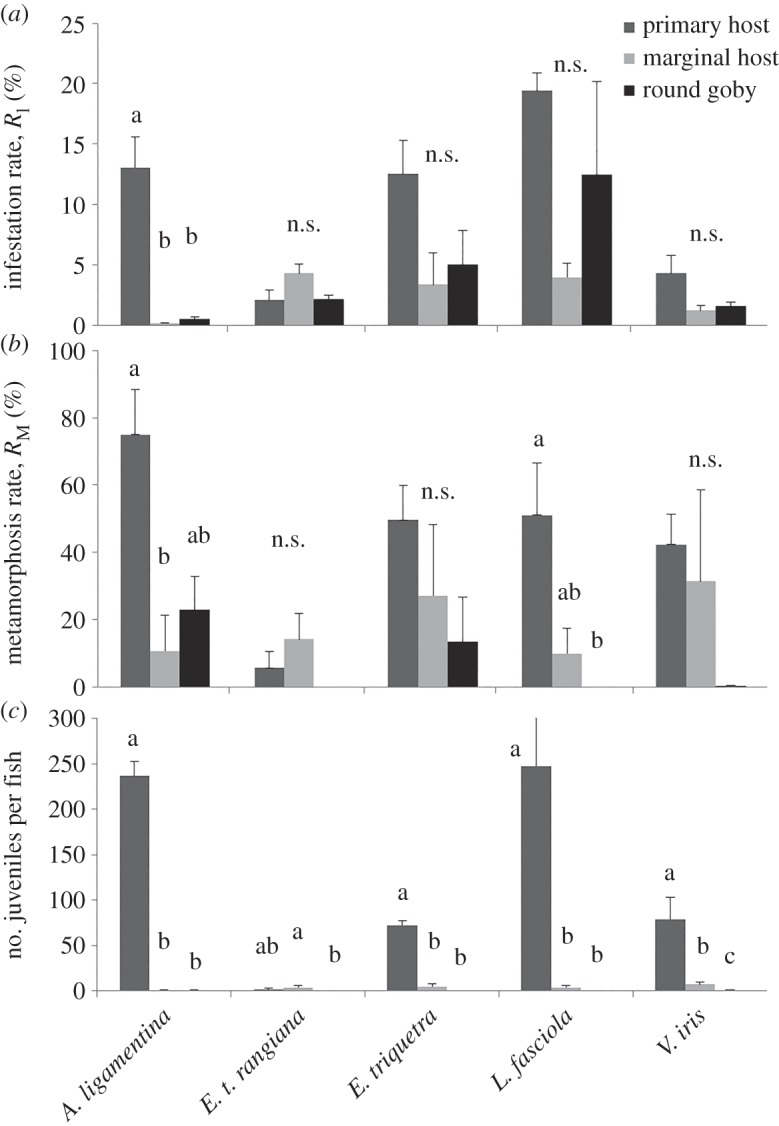
Experimental values (mean ± s.e.) for all mussel species and all host types (primary host, marginal host and round goby, *Neogobius melanostomus*). Primary hosts of: *Actinonaias ligamentina* = *Micropterus salmoides*; *Epioblasma torulosa rangiana* = *Etheostoma exile*; *Epioblasma triquetra* = *Percina caprodes; Lampsilis. fasciola* *=* *Micropterus dolomieu; Villosa iris* *=* *Ambloplites rupestris*). *Cottus bairdii* served as the marginal host for all species. ‘n.s.’ indicates non-significant differences; different letters above two host types within a mussel species indicate significant differences. (*a*) Infestation rates, (*b*) metamorphosis rates and (*c*) number of juveniles produced per fish.

Infestation (*R*_I_) and metamorphosis rates (*R*_M_) were generally highest on the primary host, followed by the marginal host and then *N. melanostomus* (figure 1*a,b*). This pattern was also seen in juvenile mussel production (figure 1*c*). Considerable variation in each of these values was observed including the number of glochidia that successfully attached to individual fish, which was revealed by dissections of fishes that died prior to completion of the experiments (mortality rate for primary hosts: 33 ± 16%; *C. bairdii*: 2 ± 2% and *N. melanostomus*: 20 ± 10%).

#### 3.1.1. Actinonaias ligamentina

Infestation rates differed significantly among fish species (Kruskall–Wallis; $\chi_{2}^{2}=6.50,$ *p* = 0.039) and were higher on the primary host (*M. salmoides*) versus the marginal host (*C. bairdii*; *p* = 0.034). Metamorphosis rates also differed (ANOVA *F*_2,4_ = 8.00, *p* = 0.040) with the highest rates on the primary host versus marginal hosts (post-hoc Tukey test, *p* = 0.044) and *N. melanostomus* (*p* = 0.064). Juvenile production did not differ significantly ($\chi_{2}^{2}=4.23,$ *p* = 0.12), but this was likely the result of reduced power due to fish mortality as differences were detected under ANOVA (*F*_2,4_ = 177, *p* = 0.0001). Ten juvenile *A. ligamentina* were produced on *N. melanostomus*.

#### 3.1.2. Epioblasma torulosa rangiana

Infestation rates did not differ significantly (*F*_2,6_ = 3.06, *p* = 0.12), but marginal differences in *R*_M_ were observed (*F*_2,6_ = 4.03, *p* = 0.078) with the highest rates on the marginal host (*C. bairdii*), followed by the primary host (*E. exile*) and *N. melanostomus.* Juvenile production differed significantly (*F*_2,6_ = 5.20, *p* = 0.049), with the highest production on the marginal host, followed by the primary host and *N. melanostomus.* Significant differences were found between the marginal host (*C. bairdii*) and *N. melanostomus* (*p* = 0.042). No juvenile *E. t. rangiana* were produced on *N. melanostomus*.

#### 3.1.3. Epioblasma triquetra

Infestation rates did not differ significantly (*F*_2,6_ = 3.11, *p* = 0.12), nor did *R*_M_ (*F*_2,6_ = 1.37, *p* = 0.32); however, the highest rates were on the primary host (*P. caprodes*), followed by the marginal host (*C. bairdii*) and then *N. melanostomus.* Juvenile production was marginally significantly different ($\chi_{2}^{2}=6.01,$ *p* = 0.05) with marginal differences between the primary host and *N. melanostomus* (*p* = 0.05). Four juvenile *E. triquetra* were produced on *N. melanostomus*.

#### 3.1.4. Lampsilis fasciola

Infestation rates did not differ significantly (*F*_2,6_ = 2.84, *p* = 0.14), whereas *R*_M_ did (*F*_2,5_ = 13.2, *p* = 0.010) with the highest rates on the primary host (*M. dolomieu*), followed by the marginal host (*C. bairdii*), and then *N. melanostomus*. Significant differences were found between the primary host and *N. melanostomus* (*p* = 0.009) and marginally with the marginal host (*p* = 0.056). Juvenile production differed significantly (*F*_2,5_ = 32.2, *p* = 0.001), with the highest production on the primary host, followed by the marginal host and *N. melanostomus*. Differences were detected between the primary host and *N. melanostomus* (*p* = 0.001) and the marginal host (*p* = 0.005). No juvenile *L. fasciola* were produced on *N. melanostomus*.

#### 3.1.5. Villosa iris

Significant differences in the viability of glochidia among female mussels were observed, so two-way ANOVAs were conducted. Infestation rates did not differ significantly among fish species or female mussels (fish factor: *F*_2,4_ = 3.24, *p* = 0.15; mussel factor: *F*_2,4_ = 0.31, *p* = 0.75). One-way ANOVAs were conducted to assess *R*_M_ and the number of juvenile mussels produced per fish as fish mortality reduced the number of replicates per female. Metamorphosis rates did not differ significantly (*F*_2,4_ = 1.49, *p* = 0.33), but the number of juveniles produced per fish did (*F*_2,4_ = 34.0, *p* = 0.003) with the highest production on the primary host (*A. rupestris*), followed by the marginal host (*C. bairdii*) and *N. melanostomus* (*p* < 0.04 for all pairwise comparisons). One juvenile *V. iris* was produced on *N. melanostomus.* There was high mortality of *N. melanostomus* with attached glochidia (0–19 per fish).

### 3.2. Natural infection of *Neogobius melanostomus* in the field

**Figure 2.**
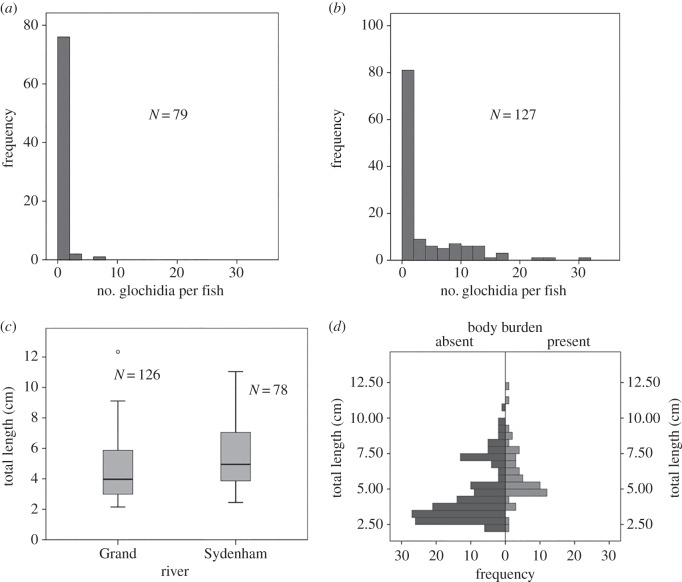
Frequency distributions of glochidia infection on *Neogobius melanostomus* in the field. Glochidia per individual *N. melanostomus* in the (*a*) Sydenham and (*b*) Grand rivers, (*c*) total lengths of *N. melanostomus* with infections from both rivers (boxes indicate the spread of total lengths; horizontal lines within the boxes indicate medians; whiskers indicate the smallest and largest values; and outliers are represented by open circles; two fish missing tails were not included) and (*d*) total lengths and the proportion of fish infected with glochidia (present or absent) of *N. melanostomus* collected from both rivers.

There was a significantly higher prevalence of glochidia infection on *N. melanostomus* in the Grand versus the Sydenham river (50/127 (39.3%) versus 4/79 (5.1%), of fish, respectively; $\chi_{1}^{2}=34.88,$ *p* < 0.001, *N* = 206 using a non-parametric median test following arcsine square root transformation; figure 2), and glochidia were only found attached to the gills. Individual fish also exhibited different intensity of glochidia infection (i.e. parasite burden), with up to 30 glochidia/fish in the Grand River versus up to 6 glochidia/fish in the Sydenham River (figure 2*a,b*). Although *N. melanostomus* from the Grand River were significantly smaller than those from the Sydenham River (Mann–Whitney *U* = 6112, *p* = 0.003, *N* = 204, two individuals were missing tails; figure 2*c*), the logistic regression of glochidia infection (i.e. 0 = not infected, 1 = glochidia infected) on TL was not significant (Nagelkerke's *R*^2^ = 0.109).

Sixteen of the glochidia found encysted on *N. melanostomus* from Grand River site GR-d (42.958570, −79.869990) and 171 of the glochidia found encysted from site GR-a (43.087610, −80.040690) were removed, measured and classified according to the DFA model as: *A. ligamentina* (*n* = 13), *Alasmidonta viridis* (*n* = 1), *Amblema plicata* (*n* = 68), *Elliptio dilatata* (*n* = 17), *Epioblasma triquetra* (*n* = 15), *Fusconaia flava* (*n* = 9), *Lasmigona complanata* (*n* = 1), *Obovaria reflexa* (*n* = 36), *Obovaria olivaria* (*n* = 5), *Pleurobema sintoxia* (*n* = 7), *Quadrula pustulosa* (*n* = 1), *Quadrula quadrula* (*n* = 2) and *Toxolasma parvum* (*n* = 12). Four glochidia removed from *N. melanostomus* at the three sites on the Sydenham River ((42.608695, –82.122231), (42.636484, –82.01992), (42.655677, –82.008247)) were similarly classified as: *A. plicata* (*n* = 1), *E. t. rangiana* (*n* = 1), *Ligumia recta* (*n* = 1) and *O. reflexa* (*n* = 1). The glochidia from as many as six unionid species were encysted on a given fish from the Grand River.

### 3.3. Modelled contribution of *Neogobius melanostomus* to unionid recruitment

**Figure 3.**
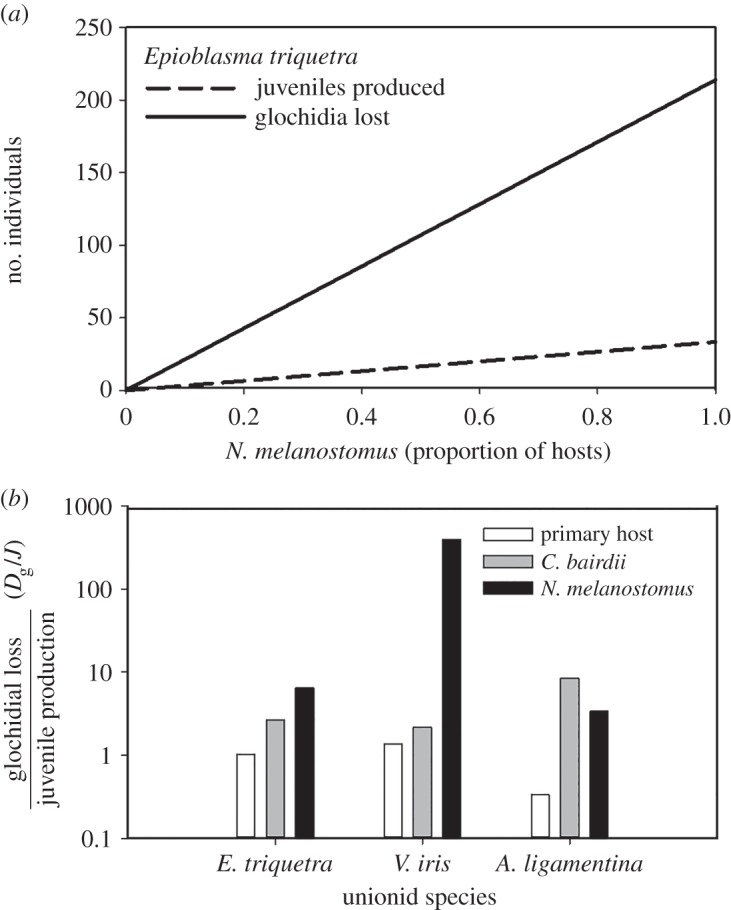
Modelled juvenile production and glochidial loss due to unionid infection on *Neogobius melanostomus* using equations (2.2) and (2.3) and the parameter values listed in the electronic supplementary material, table S1. The model could not be applied to two species whose glochidia successfully infested but failed to metamorphose on *N. melanostomus*. (*a*) Number of juvenile *Epioblasma triquetra* produced and glochidia lost as *N. melanostomus* dominated the proportion of host fish in the system. (*b*) The ratio of glochidia loss to juvenile mussel produced (i.e. *D*_g_/*J*; note log scale) on *N. melanostomus* versus primary fish hosts (*Percina caprodes* for *E. triquetra*; *Ambloplites rupestris* for *V. iris; and Micropterus salmoides* for *A. ligamentina*) and *Cottus bairdii* (a known marginal host for these unionids).

The ratio of the slopes of glochidia lost from potential recruitment (*D*_g_) to the slope of juvenile mussels produced (*J*) versus the relative abundance of *N. melanostomus* to known suitable fish hosts (*N*; proportion between 0 and 1.0) was assessed using species-specific parameter values for *E. triquetra, V. iris* and *A. ligamentina* (electronic supplementary material, table S1). The loss of glochidia by *N. melanostomus* increased 6.41× faster than juvenile production for *E. triquetra* (figure 3*a*), 394× faster for *V. iris* and 3.35× faster for *A. ligamentina* (figure 3*b*). These *D*_g_ : *J* ratios were always lowest for the primary hosts (1.01, 1.36 and 0.33, respectively, for *E. triquetra, V. iris* and *A. ligamentina*), were generally higher for the marginal host (*C. bairdii;* 2.68, 2.18 and 8.34, respectively, for *E. triquetra, V. iris* and *A. ligamentina*) and highest for *N. melanostomus* (figure 3*b*). The trend between the marginal host and *N. melanostomus* was reversed on *A. ligamentina,* likely due to the fact that very few *C. bairdii* survived until the end of the metamorphosis period, which limited the assessment of *R*_M_. In the case of *E. t. rangiana* and *L. fasciola*, none of the 164 and 1827 encysted glochidia, respectively, metamorphosed on *N. melanostomus* thus representing a complete loss of reproductive output.

## 4. Discussion

The results of this study reveal a novel way in which *N. melanostomus* affect unionid mussels. Specifically, relatively high laboratory infestation rates confirmed by the occurrence of glochidia infection on *N. melanostomus* in nature, combined with relatively low metamorphosis rates in the laboratory support the hypothesis that *N. melanostomus* are a sink for unionid glochidia. Model results indicate that *N. melanostomus* contribute more to the loss of reproductive potential in unionid mussels than to their recruitment. To the best of our knowledge, this represents a hitherto unknown manner by which an invasive species affects a native species. This system in which unionid glochidia are intercepted by an invasive host, and subsequently metamorphose at low rates or not at all, is analogous to that of native plants whose pollen is transported to incompatible invasive species and essentially wasted [26]. It differs from parasite spillback systems [28] because increased parasitism of invasive fish hosts represents a sink for glochidia and a loss to native mussel communities.

Generally, glochidial infestation rates on *N. melanostomus* were similar to those on known primary and marginal hosts; however, metamorphosis rates and juvenile mussel production were generally higher on primary hosts. Moreover, metamorphosis on *N. melanostomus* was only observed for three of the five unionid species examined (endangered *V. iris* and *E. triquetra*, and a common species, *A. ligamentina*). This indicates that although initial attachment of glochidia to *N. melanostomus* occurs, glochidia do not metamorphose at rates comparable to their primary or marginal hosts. Initial attachment is not surprising, given that glochidia attach to non-host fish [15] as well as to inanimate objects [29]. Longer term attachment and encystment, necessary for successful juvenile mussel production, requires some set of species-specific chemical cues [29] that did not occur for *N. melanostomus*.

The infestation rates on primary and marginal hosts are consistent with previous experiments that have examined these mussel-host fish using individuals from the northern limit of their respective species ranges [30] (K. Loftus 2014, Ontario Ministry of Natural Resources and Forestry, personal communication), as is the variable number of encysted glochidia on an individual fish for a given mussel–fish combination [31]. Regardless, the laboratory experiments permitted a quantitative assessment of which unionid species are able to use *N. melanostomus* as a host. This is the first study, to our knowledge, which has specifically assessed the natural glochidial infection of *N. melanostomus* in North America.

Glochidia infection rates of approximately 40% in the Grand River indicate that *N. melanostomus* encounter and become infected with glochidia at rates comparable to known hosts: infection rates of 34% have been reported on *M. dolomieu* by *L. fasciola* or another *Lampsilis* species (Morris and Granados 2010, Fisheries and Oceans Canada, personal communication); infection rates of 40% were found on *Morone americana*, which is a host of *Leptodea ochracea*, and *Lampsilis cariosa* [32]; and infection rates of 46–71% were found on *P. caprodes* and *A. rupestris*, which are known hosts of some rare unionid mussels (D. Woolnough 2011, Central Michigan University, personal communication). The reduced infection rates observed in the Sydenham River may be reflective of the recent invasion of this system by *N*. *melanostomus* as subsequent samples obtained in 2013 and 2014 have shown glochidia infection rates of 36% (29 of 79 fish) and 81% (50 of 61 fish) (T.J.M. 2015, personal communication).

Although the spatial and temporal nature of the unionid–*N. melanostomus* encounters is not known, the analysis of *N. melanostomus* from the Grand River provides some valuable information. The majority of encysted glochidia included *A. ligamentina*, *A. plicata, E. dilatata*, *E. triquetra* and *O. reflexa*. *Actinonaias ligamentina*, the common species examined in the laboratory experiments, is a host generalist that broadcasts its glochidia into the water column [33], so glochidia encounter with *N. melanostomus* is not surprising. *Amblema plicata* is also a host generalist [33] and is common. *Elliptio dilatata,* which is common in Ontario (S Rank = 5 [34]) and a host generalist [33], is not abundant in the lower Grand River where *N. melanostomus* were collected [35]. Conversely, *E. triquetra* and *O. reflexa* are host specialists [33] and rare in Ontario [6]. Interestingly, *O. reflexa* release glochidia in worm-like conglutinates (i.e. packages of glochida formed by the female mussel) [33] and *A. plicata* release fish-like conglutinates. A benthic fish such as *N. melanostomus* would likely consider conglutinates as prey items and hence become infested. There may be an association between glochidial dispersal/host attraction mechanism and infection on *N. melanostomus*, which suggests that the effect of *N. melanostomus* on unionid species may not be uniform across the taxon. This warrants additional study.

The relative contribution of *N. melanostomus* to juvenile mussel production (*J*) versus glochidia loss (*D*_g_) is determined primarily by the metamorphosis rate (*R*_M_) of glochidia on *N. melanostomus* (equation (2.3)/(2.2)) given by

4.1 $$\frac{D_{g}}{J}=\frac{1-R_{M}}{R_{M}}.$$

Both *J* and *D*_g_ increase as the proportion of *N. melanostomus* increase, but higher *R*_M_ leads to more *J*. This model does not account for differences in host attraction strategies among unionids [9], nor predation or competition for conglutinates with natural host fishes by *N. melanostomus*, which could result in *N. melanostomus*-mediated recruitment being the sole source of *J* for some species whose hosts experience decline or are extirpated. *Neogobius melanostomus* may reduce the pool of effective hosts for unionids where they co-occur, interfere with the normal reproductive cycle of unionids when they achieve high population densities [16], and consequently have a greater impact on these biodiversity ‘hot-spots' than was predicted [5]. Such indirect effects have been recently demonstrated in *Anadonta anatina* (a host generalist), which had lower metamorphosis on non-native fish hosts and lower success overall (i.e. 67–94% fewer fish species) as a result of ‘biotic homogenization’ [16]. Although there is error associated with our model, especially the error associated with estimate of *R*_M_ for relatively poor host species, and simplification of the mussel–host interactions in the laboratory, the implication of glochidia loss compared with primary hosts remains a fundamental finding, which will be exacerbated as *N. melanostomus* dominates the ecosystem in biodiversity hot-spots. It is relevant to note that significant differences in infection rates have been found between recently isolated mussel populations [36], and such differences may also occur between geographically distinct lineages of host fish as well as within lineages of mussels and their fish hosts [37]. Consequently, it is possible that the effects of invasive *N. melanostomus* on host–parasite relationships may vary spatially and temporally due to population-specific attributes arising from local adaptations and fine-scale coevolutionary dynamics.

The introduction of a new species into an ecosystem has the potential to cause many unanticipated effects as a result of the high variability in the rates of spread, types of impacts and species interactions [38]. The results of this study indicate that *N. melanostomus* are likely acting as a sink for glochidia, whereby they prevent glochidia from reaching their intended hosts. This has negative implications for unionid species that exhibit high rates of infection and poor/no metamorphosis on *N. melanostomu*s, particularly those species whose populations are limited geographically to areas with large populations of this invasive fish (e.g. riverine refugia of the Laurentian Great Lakes). A thorough understanding of the effects of a species invasion on an ecosystem can help to predict the effects of its invasion elsewhere, at least in terms of type and direction [39]. These predictions, in turn, dictate how much effort should be directed towards halting the invasion and spread of a new species, and more generally, contribute to our understanding of the ecology of invasions.

## Supplementary Material

Species specific values for the model of glochidia loss versus juvenile mussels produced

## Data accessibility

The supporting data are available on Dryad: http://dx.doi.org/10.5061/dryad.1kv63.

## References

[1] Munawar M, Munawar IF, Mandrak NE, Fitzpatrick M, Dermott R, Leach J 2005 An overview of the impact of non-indigenous species on the food web integrity of North American Great Lakes: Lake Erie example. Aquat. Ecosyst. Heal. Manag. 8, 375–395. (doi:10.1080/14634980500411606)

[2] Nalepa TF, Schloesser DW (eds) 2014 Quagga and zebra mussels: biology, impacts, and control, 2nd edn Boca Raton, FL: CRC Press.

[3] Mackie G 1991 Biology of the exotic zebra mussel, *Dreissena polymorpha*, in relation to native bivalves and its potential impact in Lake St. Clair. Hydrobiologia 219, 251–268. (doi:10.1007/BF00024759)

[4] Nalepa TF, Hartson DJ, Gostenik GW, Fanslow DL, Lang GA 1996 Changes in the freshwater mussel community of Lake St. Clair: from Unionidae to *Dreissena polymorpha* in eight years. J. Great Lakes Res. 22, 354–369. (doi:10.1016/S0380-1330(96)70961-9)

[5] Poos M, Dextrase A, Schwalb AN, Ackerman JD 2010 Secondary invasion of the round goby into high diversity Great Lakes tributaries and species at risk hotspots: potential new concerns for endangered freshwater species. Biol. Invasions 12, 1269–1284. (doi:10.1007/s10530-009-9545-x)

[6] COSEWIC. 2014 Wildlife Species Search. Database: Committee on the Status of Endangered Wildlife in Canada.

[7] Jude DJ, Janssen J, Crawford G 1995 Ecology, distribution and impact of the newly introduced round and tubenose gobies on the biota of the St. Clair and Detroit rivers. In The Lake Huron ecosystem: ecology, fisheries and management (eds Munawar M, Edsall T, Leach J). Ecovision World Monograph Series The Hague, The Netherlands: SPB Academic Publishing.

[8] French JRP, Jude DJ 2001 Diets and diet overlap of nonindigenous gobies and small benthic native fishes co-inhabiting the St. Clair River, Michigan. J. Great Lakes Res. 27, 300–311. (doi:10.1016/S0380-1330(01)70645-4)

[9] Barnhart MC, Haag WR, Roston WN 2008 Adaptation to host infection and larval parasitism in Unionoida. J. North Am. Benthol. Soc. 27, 370–394. (doi:10.1899/07-093.1)

[10] Haag WR 2012 North American freshwater mussels: natural history, ecology, and conservation. Cambridge, UK: Cambridge University Press.

[11] McNichols KA, Mackie GL, Ackerman JD 2011 Host fish quality may explain the status of endangered *Epioblasma torulosa rangiana* and *Lampsilis fasciola* (Bivalvia: Unionidae) in Canada. J. North Am. Benthol. Soc. 30, 60–70. (doi:10.1899/10-063.1)

[12] Watters GT, Menker T, Thomas S, Kuehnl K 2005 Host identifications or confirmations. Ellipsaria 7, 11–12.

[13] Muzzall PM, Peebles CR, Thomas M 1999 Parasites of the round goby, *Neogobius melanostomus*, and tubenose goby, *Proterorhinus marmoratus* (Perciformes: Gobiidae), from the St. Clair River and Lake St. Clair, Michigan. J. Helminthol. Soc. Washingt. 62, 226–228.

[14] Kornis MS, Mercado-Silva N, Vander Zanden MJ 2012 Twenty years of invasion: a review of round goby *Neogobius melanostomus* biology, spread and ecological implications. J. Fish Biol. 80, 235–285. (doi:10.1111/j.1095-8649.2011.03157.x) 22268429 10.1111/j.1095-8649.2011.03157.x

[15] Jansen W, Bauer G, Zahner-Meike E 2001 Glochidial mortality in freshwater mussels. In Ecology and evolution of the freshwater mussels Unionoida (eds Bauer G, Wachtler K), pp. 185–211. Berlin, Germany: Springer.

[16] Douda K, Lopes-Lima M, Hinzmann M, Machado J, Varandas S, Teixeira A, Sousa R 2013 Biotic homogenization as a threat to native affiliate species: fish introductions dilute freshwater mussel's host resources. Divers. Distrib. 19, 933–942. (doi:10.1111/ddi.12044)

[17] Dodd BJ, Barnhart MC, Rogers-Lowery CL, Fobian TB, Ronald VJ 2005 Cross-resistance of largemouth bass to glochidia of unionid mussels. J. Parasitol. 91, 1064–1072. (doi:10.1645/GE-511R.1) 16419750 10.1645/GE-511R.1

[18] Huebner JD, Pynnonen KS 1992 Viability of glochidia of two species of *Anodonta* exposed to low pH and selected metals. Can. J. Zool. 70, 2348–2355. (doi:10.1139/z92-315)

[19] ASTM. 2012 Standard guide for conducting laboratory toxicity tests with freshwater mussels (E2455-06). Annu. B. ASTM Stand, E2455–05.

[20] Kidd BT 1973 Unionidae of the Grand River drainage. MSc thesis, Carleton University, Ottawa, Ontario, Canada.

[21] Staton SK, Dextrase A, Metcalfe-Smith JL, Di Maio J, Nelson M, Parish J, Kilgour B, Holm E 2003 Status and trends of Ontario's Sydenham River ecosystem in relation to aquatic species at risk. Environ. Monit. Assess. 88, 283–310. (doi:10.1023/A:1025529409422) 14570419 10.1023/a:1025529409422

[22] Zar JH 1999 Statistical analysis. Englewood Cliffs, NJ: Prentice Hall.

[23] Quinn GP, Keough MJ 2002 Experimental design and data analysis for biologists. Cambridge, UK: Cambridge University Press.

[24] Tremblay MEM, Morris TJ, Ackerman JD 2015 A multivariate approach to the identification of unionid glochidia with emphasis on Species at Risk in Southern Ontario. Can. Manuscr. Rep. Fish Aquat. Sci. 3057, vii + 52.

[25] McNichols KA 2007 Implementing recovery strategies for mussel species at risk in Ontario. MSc thesis, University of Guelph, Ontario, Canada.

[26] Bjerknes A, Totland O, Hegland SJ, Nielsen A 2007 Do alien plant invasions really affect pollination success in native plant species? Conserv. Biol. 138, 1–12. (doi:10.1016/j.biocon.2007.04.015)

[27] Schwalb AN, Garvie M, Ackerman JD 2010 Dispersion of freshwater mussel larvae in a lowland river. Limnol. Oceanogr. 55, 628–638. (doi:10.4319/lo.2009.55.2.0628)

[28] Kelly DW, Paterson RA, Townsend CR, Poulin R, Tompkins DM 2009 Parasite spillback: a neglected concept in invasion ecology? Ecology 90, 2047–2056. (doi:10.1890/08-1085.1) 19739367 10.1890/08-1085.1

[29] Wood EM 1974 Some mechanisms involved in host recognition and attachment of the glochidium larva of *Anodonta cygnea* (Mollusca: Bivalvia). J. Zool. 173, 15–30. (doi:10.1111/j.1469-7998.1974.tb01744.x)

[30] Barnhart MC, Baird MS 2000 Fish hosts and culture of mussel species of special concern: annual report for 1999. Columbus, MO: US Fish and Wildlife Service and Missouri Department of Conservation.

[31] Riusech FA, Barnhart MC 2000 Host suitability and utilization in *Venustaconcha ellipsiformis* and *Venustaconcha pleasii* (Bivalvia:Unionidae) from the Ozark Plateaus. In *Proc. Conservation, Captive Care and Propagation of Freshwater Mussels Symp. 1998*, pp. 83–91. Columbus, OH: Ohio Biological Survey.

[32] Kneeland SC, Rhymer JM 2008 Determination of fish host use by wild populations of rare freshwater mussels using a molecular identification key to identify glochidia. J. North Am. Benthol. Soc. 27, 150–160. (doi:10.1899/07-036.1)

[33] Watters GT, Hoggarth MA, Stansbery DH 2009 The freshwater mussels of Ohio. Columbus, OH: Ohio State University Press.

[34] Cordeiro J 2009 Comprehensive Report Species *Elliptio dilatata*. Database: NatureServe.

[35] Metcalfe-Smith JL, Mackie GL, Di Maio J, Staton SK 2000 Changes over time in the diversity and distribution of freshwater mussels (Unionidae) in the Grand River. J. Great Lakes Res. 26, 445–459. (doi:10.1016/S0380-1330(00)70707-6)

[36] Douda K, Sell J, Kubíková-Peláková L, Horky P, Kaczmarczyk A, Mioduchowska M 2014 Host compatibility as a critical factor in management unit recognition: population-level differences in mussel–fish relationships. J. Appl. Ecol. 51, 1085–1095. (doi:10.1111/1365-2664.12264)

[37] Reichard M et al. 2015 Population-specific responses to an invasive species. Proc. R. Soc. B 282, 20151063 (doi:10.1098/rspb.2015.1063) 10.1098/rspb.2015.1063 PMC4528524 26180070

[38] Melbourne BA, Hastings A 2009 Highly variable spread rates in replicated biological invasions: fundamental limits to predictability. Science 325, 1536–1539. (doi:10.1126/science.1176138) 19762641 10.1126/science.1176138

[39] Kulhanek SA, Ricciardi A, Leung B 2011 Is invasion history a useful tool for predicting the impacts of the world's worst aquatic invasive species? Ecol. Appl. 21, 189–202. (doi:10.1890/09-1452.1) 21516897 10.1890/09-1452.1

