# Supplementary material for: Loss of reproductive output caused by an invasive species
Source: R Soc Open Sci. 2016 Apr 6;3(4):150481. doi: 10.1098/rsos.150481 (PMC4852625; doi:10.1098/rsos.150481)
Supplement: Species specific values for the model of glochidia loss versus juvenile mussels produced [file rsos150481supp1.docx]

Table S1. Species‑specific values for the model of glochidia loss versus juvenile mussels produced (all values correspond to the Sydenham River). Sources: McNichols 2007, Schwalb et al. 2010, COSEWIC, present study.

| **Fish Species** | **Parameter** | **Species** | | |
| --- | --- | --- | --- | --- |
|  |  | ***E. triquetra*** | ***V. iris*** | ***A. ligamentina*** |
|  | Occupied area (m^2^) | 25,000 | 630,000 | 1,650,000 |
|  | (% occupied **×** total area) |  |  |  |
|  | Density of mussels (no./m^2^) | 0.09 | 0.03 | 0.69 |
|  | Number of mussels | 2,250 | 18,900 | 1,142,343 |
|  | (density **×** area occupied) |  |  |  |
|  | *U* = number of females | 1,125 (2.5:1 M:F) | 9,450 ^a^ | 571,171 ^a^ |
|  | *f* = fecundity | 17,580 | ~25,000 ^b^ | 1,000,000 |
|  | (glochidia/female) |  |  |  |
|  | *R_e_* (encounter rate) = (10^-2^–10^-4^) | 0.001 | 0.001 | 0.001 |
| *N. melanostomus* | Infestation rate per tank (%) ^c^ | 4.98 | 1.57 | 0.48 |
|  | *R_I_*, infestation rate per fish (%) | 1.25 | 0.522 | 0.12 |
|  | *R_M_*, metamorphosis rate (%) | 13.5 | 0.253 | 23.0 |
| *C. bairdii* | Infestation rate per tank (%) ^c^ | 3.35 | 1.97 | 0.15 |
|  | *R_I_*, infestation rate per fish (%) | 0.837 | 0.655 | 0.0375 |
|  | *R_M_* metamorphosis rate (%) | 27.18 | 31.4 | 10.7 |
| Primary Host ^d^ | Infestation rate per tank (%) ^c^ | 12.5 | 4.27 | 13.1 |
|  | *R_I_*, infestation rate per fish (%) | 3.125 | 1.0675 | 3.275 |
|  | *R_M_* metamorphosis rate (%) | 49.7 | 42.3 | 75.0 |

^a^ assumed 1:1 M:F, ^b^ estimate, ^c^ attached glochidia/total glochidia, ^d^ primary fish hosts: *Percina caprodes* for *E. triquetra*; *Ambloplites rupestris* for *V. iris; and Micropterus salmoides* for *A. ligamentina.*
